# The hidden sentinel of the skin: An overview on the role of interleukin-13 in atopic dermatitis

**DOI:** 10.3389/fmed.2023.1165098

**Published:** 2023-04-18

**Authors:** Maddalena Napolitano, Francesca di Vico, Angelo Ruggiero, Gabriella Fabbrocini, Cataldo Patruno

**Affiliations:** ^1^Department of Medicine and Health Sciences Vincenzo Tiberio, University of Molise, Campobasso, Italy; ^2^Section of Dermatology, Department of Clinical Medicine and Surgery, University of Naples Federico II, Naples, Campania, Italy; ^3^Department of Health Sciences, University Magna Graecia of Catanzaro, Catanzaro, Italy

**Keywords:** atopic dermatitis (AD), interleukin-13, pathogenesis, type 2 inflammation, review

## Abstract

Recent evidence suggests that interleukin (IL)-13 is a crucial cytokine involved in the pathogenesis of atopic dermatitis (AD). It is a central driver of type-2 T-helper inflammation and is overexpressed in lesional skin of AD patients. Upon release in peripheral skin, IL-13 activates its receptors, recruits inflammatory cells, and modifies the skin microbiome. IL-13 also reduces the expression of epidermal barrier proteins and activates sensory nerve mediating the itch transmission signal. Novel therapeutics that target IL-13 seem to be efficacious and safe for the treatment of patients with moderate-to-severe AD. The aim of our manuscript is to review the role that IL-13 plays in AD immunopathogenesis.

## Introduction

Atopic dermatitis (AD) is a chronic inflammatory skin disease which has a complex and not yet fully understood pathophysiology (1, 2). AD is heterogeneous in its onset age, clinical phenotype, and severity of manifestations (1, 2). It has been reported that, in developed countries, AD affects in lifetime up to one fifth of the general population, resulting the most frequent among chronic inflammatory skin diseases (3–6). AD, especially in its moderate-to severe forms, results in a negative impact on the quality of life (QoL) of both patients, but and their families (6). Itch is the most troublesome symptom and has been linked with sleep disturbance, reduced work productivity, and poor mental health (7).

Atopic dermatitis pathophysiology implicates multiple interactions between altered type 2 immune responses, skin microbiome dysbiosis, and epidermal barrier disruption (EBD) (8). Recent research advancements, lead to a crucial change in AD pathogenic model, overcoming the preceding hypotheses based on (i) key role of the EBD (“outside-in” theory); (ii) immunoglobulin E (IgE)- response (type 1 hypersensitivity), or (iii) principal role of the abnormal systemic immune activation (“inside-out” theory) (9–11). Currently, however, AD is thought to be secondary to the complex interaction between several genetic defects, environmental stimuli and the activation of intricate inflammatory pathways that induce both the onset and chronicity of the disease (8–11). The discovery of the role for these multiple immune pathways and of related cytokines has led to the development of new drugs (8).

Atopic dermatitis is a disease related to type 2 immunity activation in response to environmental stimuli (2). Both adaptive and innate immune systems are involved in Type 2 immunity (2, 12). Indeed, in the innate immune system activation, it has been showed that key roles are played by Group 2 lymphoid cells (ILC2), mast cells, basophils, eosinophils, and macrophages activated by interleukin (IL)-4 and/or IL-13 (12–15). Furthermore, also keratinocyte is implicated in this mechanism by expressing an increased level of IL-25, IL-33, and thymic stromal lymphopoietin (TSLP) (12–15). These cytokines may act as alarmins, inducing the production of IL-4, IL-13, and IL-5 from both T helper (Th)2, and ILC2 cells, which in turn trigger the complex inflammatory cascade that underlies the clinical expression of AD (16).

Additionally, adaptive immune responses of T and B cells, including CD4 Th2 cells and Th17 cytokines, are required for the development and progression to systemic inflammation in the atopic march (16). The activation of Th2 and ILC2 pathways are at the center of type 2 inflammation (12–15). Conversely, although the expression of Th17-cytokines such as IL-17A has consistently been found to be increased in AD lesions, the role of these cytokines in AD pathogenesis is controversial (16). It has been supposed that they have a role in AD inflammation, rather, its presence is part of an effector response against Staphylococcus aureus (16).

In particular, IL-4 and IL-13 play a key role in AD pathogenesis, orchestrating effector Th2 immune responses (12). Even if IL-4 and IL-13 are encoded by adjacent genes and share a common receptor and signaling pathway, they are differently expressed *in vivo* by a variety of distinct cells that control both innate and adaptive immunity (12). Indeed, the follicular Th cells of the lymph node, regulating B-cell immunity, invariant natural killer T2 cells, and basophils express IL-4, while mucosal ILC2s mostly express IL-13 and little IL-4 (17). This difference suggests that IL-13 and IL-4 may have distinct actions in Th2 immunity (12). Indeed, it has been showed that IL-4, which drives the T cell differentiation, may have a role in the first steps of the pathogenesis of AD, while IL-13 effects appear to influence the peripheral tissue cells and the effector phase of the immune response (12–17). The aim of this narrative review is to provide an overview of the role that IL13 has in AD pathogenesis.

## Methods

For this narrative review of literature, search of the English-language literature regarding the pathogenic role of IL-13 in AD was conducted. Different databases, namely Embase, PubMed, ResearchGate, Google Scholar and Scopus, have been consulted using the following terms: interleukin 13, IL-13, atopic dermatitis, atopic eczema, pathogenesis, pathogenic mechanism, Th2 inflammation.

## IL-13 characteristics and signaling

IL-13 is an immunoregulatory cytokine with a structure characterized by a 4 α-helical hydrophobic bundle core (18). Although it is mainly secreted by Th2 cells, other cells, such as ILC2, mast cells and basophils, release IL-13 (4, 14).

A subgroup of ILCs, the ILC2, which are the only subset of lymphocytes with no antigen receptors rearranged, produce type 2 cytokines, promoting inflammation and hyperresponsiveness (18–21). In the skin, dermal ILC2s secrete IL-13 regardless of allergen exposure and independently of the alarmins (IL-2, TSLP, and IL-33), driving dendritic cells (DC)2 precursors differentiation to a CD11b^low^ phenotype that fosters Th2 priming (12). Conversely, in the lung ILC2s require alarmins to secrete IL-13 and induce the expression of Th2 cells (12). However, TSLP may directly stimulate CD11c + DCs differentiation and activation, resulting in the stimulation of Th2-cell expression, inhibition of IL-12 secretion in the context of type-2 innate ligands, affecting B-cell development and survival (21). Recently, it has been demonstrated that the level of resident group ILC2 of the healthy human skin is notably increased in AD skin lesions (22). It has been showed that ILC2 in AD skin exhibits an elusive immunophenotype, and that among all the ILCs, inducible T-cell costimulatory (ICOS)-expressing cells, comprising both ILC2 and ILC3, are the main producer of IL-13 in the dermis (23). Moreover, ILCs present a higher level of IL-13Rα1 subunit of IL-13 receptor (IL-13R) than T cells (23).

The other major source of IL-13 is Th2 cells (4). In *ex vivo* coculture model, it has been demonstrated that a cutaneous lymphocyte-associated antigen (CLA)-dependent production of IL-13, upon activation with staphylococcal enterotoxin B (SEB), allows the differentiation of both Th2 high and low responder groups (24). SEB activation of the CLA T-cells resulted in a predominant IL-13 production among the Th2 cytokines (IL-5, IL-4, lL-13), and in stratification into the Th2 high and Th2 low groups, corresponding with disease activity (24). Additionally, in the Th2 high group, IL-13 response directly correlates with AD severity [measured by Eczema Area Severity Index (EASI)], anti-Staphylococcus aureus IgE plasma levels, sIL-2R, and CCL17 (24). Regardless of source, the increased IL-13 expression leads to the recruitment of eosinophils and activated T cells, resulting in an amplification of skin IL-13/IL-4 expression, driving the pathway of chronic inflammation in AD (25).

The signaling of IL-13 is regulated by a complex receptor system (4, 14). In non-hematopoietic cells, IL-13 engages a heterodimeric receptor composed of 2 subunits, the alpha chain of the IL-4 receptor (IL-4Rα) and the alpha-1 chain of IL-13 (IL-13 Rα1), binding IL-13 with low affinity; however, after forming a complex with IL-4Rα, it shows a higher affinity, resulting in the induction of the effector functions of IL-13 (4, 14). Another receptor, composed by IL-13-specific binding chain alpha 2, named IL-13 Rα2, is strictly related to IL-13 Rα1. IL-13 Rα2 binds IL-13 with high affinity, and it is considered for having a compensatory role (decoy receptor), as it would be able to remove excess IL-13 (4, 14). The IL-13 binding of the functional heterodimeric IL-4Rα /IL-13 Rα1 receptor results in the activation of downstream tyrosine kinase 2 (TYK2), and Janus kinases (JAK) activating the signal transducer and activator of transcription (STAT3, STAT1 and STAT6) (4, 14). Thus, the activation of JAK–STAT pathway is followed by the increased secretion of several chemokines for eosinophils, cytokines, angiogenic factors, and growth of IgE level binding to mast cell receptors, resulting in the exacerbation of the inflammation process of AD (25).

## Levels of expression of IL-13 in atopic skin

Previous studies showed the key role played by IL-13 in AD pathogenesis, regarding multiple aspects of disease pathogenesis such as skin barrier disruption, epidermal thickening, itch, inflammation, and infection (26). In biopsies AD skin, there is an overexpression of IL-13 in both lesional and non lesional skin compared to healthy controls (27). Additionally, AD severity is directly linked to the increased skin expression of IL-13, while a decrease in its concentration has been shown to correlate to improved clinical outcomes (27). Furthermore, a recent study confirms the dominance of the expression of IL-13 mRNA in both chronic and subacute lesions than non-lesional skin, and than healthy controls with near undetectable IL −4 expression (28–30). Recently, IL-13 has been identified in skin samples as a biomarker of AD, showing a strongest association with disease severity, circulating eosinophil levels, and total serum IgE (31). Furthermore, several atopic stigmata were associated with high levels of cutaneous IL13 such as thinning of the lateral eyebrow (Hertoghe sign), and maternal atopic rhinitis (31).

## Effects of IL-13 on skin barrier

The STAT6 activation, by IL-4 and IL-13, reduce the expression of structural proteins like hornerin, desmoglein, loricrin, involucrin, desmocollin, filaggrin (FLG), keratin 1, and keratin 10, as well as the lipid composition (*ceramides, free fatty acids, and cholestero*l) important for normal skin barrier function (27, 32, 33). The overexpression of these cytokines, therefore, plays a key role in maintaining and increasing the impairment of the skin barrier in AD (34). Additionally, they induce a decreased production of AMP by keratinocytes, thus being important also in inducing skin dysbiosis, which is characterized by a significant colonization with Staphylococcus aureus, which may precede the onset of AD lesions (34).

OVOL1, an upstream transcription factor, regulates the expression of FLG (17); OVOL1 activation leads to its cytoplasmic-to-nuclear translocation, and resulting in the up-regulation of loricrin and FLG expression (35, 36). Interestingly, IL-13 and IL-4 inhibit FLG expression by interfering with OVOL1 signaling (16). Moreover, IL-13 inhibits the expression of involucrin in an OVOL1-independent way, exacerbating barrier dysfunction (35, 36). In case of barrier-disrupted skin, keratinocytes produce high levels of IL-33, IL-25 and TSLP, promoting Th2 cells differentiation, and ILC2s, resulting in the stimulation of IL-13 production (37). Thus, a vicious cycle is formed to develop atopic dry skin. These findings suggest that IL-13–OVOL1–FLG axis may play a central role in the pathogenesis of AD (17). Near this axis, IL-13-induced FLG down-regulation is in part mediated by the IL13–periostin–IL-24 axis (17, 38). It has been showed that, through STAT6 activation, IL-13 up-regulates the keratinocytes expression of periostin, stimulating keratinocyte production of IL-24, which down-regulates the FLG expression *via* STAT3 activation (38).

Furthermore, IL-13, acting on keratinocytes, reduces the expression of skin barrier proteins and lipids, regulating the expression of metalloproteinase (MMP)-9; MMP-9 mediates tissue remodeling and the migration of cells, through action of degradation on collagen IV of the basement membrane (39, 40). Moreover, IL-13 down-regulates the expression of MMP-13 in dermal fibroblasts, leading in a decrease of the degradation of collagen, resulting in fibrosis, as found in the thickened dermis of chronic lichenified AD manifestations (41).

## Effects of IL-13 on itch

Intradermal injection of IL-13 has been reported to induce allokinesis and itching (42). The sensory neurons and keratinocytes express IL-4Rα/IL-13Rα1 and IL-13Rα2 (43). After binding to its receptor IL-13Rα1, IL-13 activates sensory neurons acting as an enhancer of other stimuli such as histamine (44). However, IL-13 is a potent neuroactive cytokine that potentiates also the responses of nonhistaminergic itch pathways (45). For example, it is involved in a histamine-independent direct stimulation of afferent nerve endings mediated by transient receptor potential ankyrin 1 (TRPA1) pathway (45).

Further, scratch injury enhanced the expression of IL-13Rα2, while no significant modification were found in the functional heterodimeric IL-13 receptor IL-13Rα1 expression (46). IL-13 Rα2, a decoy receptor binding with high affinity to IL-13, internalizes IL-13 and lowers the IL-13 level in the milieu; however, this receptor showed to have several other functions, especially in AD pathogenesis (47–51). IL-13 and scratch injury upregulate the expression of IL-13 Rα2 (47). which binds to IL-13 with high affinity, internalizes it, and decreases IL-13 levels in atopic skin (47).

Some authors also reported that IL-13 signals through IL-13 Rα2 is able to induce transforming growth factor beta (TGF-β) and promote fibrosis (48). It has been showed that even chitinase 3-like 1 (CHI3L1), also known as breast regression protein 39 (BRP-39) in mice and human homologue YKL-40, may act as an activator of IL-13 Rα2 (49, 50). The levels of both cutaneous and serum CHI3L1/YKL-40 are increased in AD patients (49). Notably, some variants of the CHI3L1 gene that codes for this protein have been associated with cases of severe AD with onset during late childhood, and a tendency to become chronic (51).

## Summary of IL-13 in atopic dermatitis

**Figure 1:**
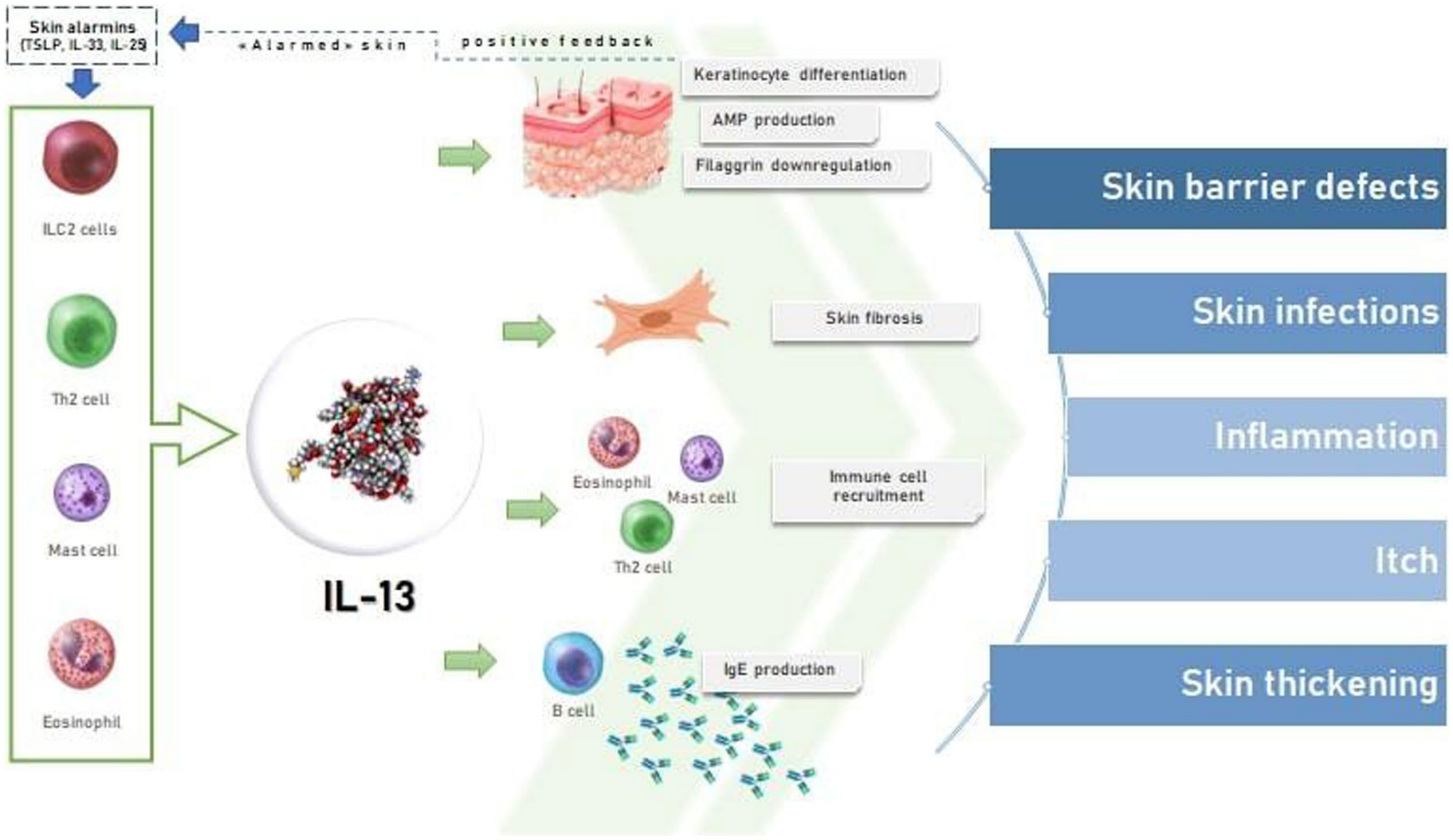
The effects of IL-13 on skin in atopic dermatitis.

IL-13 is produced from ILC2s and Th2 cells and is increased in skin from AD patients (Figure 1) (27). IL-13 binds to subunit IL-13Rα1 of its heterodimeric receptor for signaling *via* JAK1 and JAK2 (4, 14). The binding of IL-13 to IL-13Rα2, lead to the association with YKL40; the exact signaling transducing machinery related to this complex has not been fully understood (27). IL-13 contributes to the start of AD and itching and, acting with IL-4, lead to the aggravation of EBD by downregulating involucrin and FLG, *via* inactivation of OVOL1 and stimulation of periostin and IL-24 (Figure 1) (17). This contributes to the increased trans epidermal water loss in AD patients (52, 53). Moreover, IL-13 showed to decrease antimicrobial peptides (AMPs) production by keratinocytes and to have a role in the dysbiosis of the skin, characterized by a prevalent Staphylococcus aureus colonization (Figure 1) (34, 54). Hence, the increased permeability of the skin lead to the entry of antigens that reach DC, stimulating the activation of naive T cells to Th2 lymphocytes, and finally resulting in the amplification of this loop mechanism (55). Both cytokines enhance the differentiation of B-cell and the production of IgE, Th2 development/differentiation, eosinophil recruitment, hence, the amplification of the inflammation mediated by Th2 cells (55). Furthermore, in the skin, dermal ILC2s secrete IL-13 regardless of allergen exposure and independently of the alarmins driving the differentiation to Th2 cells (12). IgE binding to both basophils and mast cells, lead to an increment of histamine and other inflammatory mediators release, increasing pruritus and vasodilation (55). Stimulating action on itching, however, mainly results from a histamine-independent direct stimulation of afferent nerve endings mediated by TRPA1 pathway (55). Indeed, IL-13 is an effective, neuroactive cytokine able to modulate human sensory neurons in their neuronal excitability, increasing the itch pathways response (56). Scratching also upregulates the expression of decoy receptor IL-13Rα2 that binds to IL-13 with high affinity, internalizes it, and lowers the IL-13 level in atopic skin. Thus, if on the one hand scratching exacerbates skin inflammation, on the other it triggers a reactive compensatory response against excess levels of IL-13 by upregulating the decoy IL-13 Rα2 (47). Moreover, IL-13 also plays a role in the maintenance of conjunctival well-being, hence, its inhibition may set the premises for dupilumab induced conjunctivitis. Interestingly, this appears to be associated with eosinophil fluctuations (which are on their turn the result of diminished tissue infiltration) (57).

## Therapeutic implications

The increased knowledge of the role played by IL-4 and IL-13 in the pathogenesis of AD, resulted in the development of new targeted therapies (58). Dupilumab, the first approved biological agent for the treatment of AD, which acts by blocking IL-4Rα and thus the activity of both IL-4 and IL-13, demonstrated the effectiveness of blocking type 2 cytokines, their receptors, or their intracellular signal transducers JAK/STAT pathway (8, 59–61).

Furthermore, IL-13 signaling alone is considered a potential therapeutic target for the management of AD (58). Tralokinumab, a fully human monoclonal antibody binding IL-13, acts by preventing the binding of IL-13 to both IL-13Rα1 and IL-13Rα2, resulting in the blockage of its signal transduction, showed to be a safe as and effective treatment option, even in monotherapy in adult AD patients (52, 58, 62, 63). A phase III, double-blind, placebo plus topical corticosteroids (TCS) controlled clinical trial, in which patients were randomized 2:1 to tralokinumab 300 mg or placebo every 2 weeks (Q2W) with TCS as needed over 16 weeks (63). At week 16, an higher rate of patients treated with tralokinumab than placebo achieved EASI 75: 56% vs. 35.7% [20.2% (9.8–30.6); *p* < 0.001], and Investigator Global Assessment (IGA) 0/1: 38.9% vs. 26.2% [12.4% (2.9–21.9); *p* = 0·015] (63). Of the patients who were tralokinumab responders at week 16, 89.6 and 92.5% of those treated with tralokinumab Q2W maintained an IGA 0/1 and EASI 75 response at week 32, respectively (63). Among patients not achieving EASI 75 and IGA 0/1 with tralokinumab Q2W after 16 weeks of treatment, 55.8 and 30.5% and achieved these endpoints, respectively, after 32 weeks of treatment (63). In the ECZTRA 6 trial, a phase III study, adolescents aged between 12 and 17 years old were randomized 1:1:1 to tralokinumab 150 mg or 300 mg Q2W, or placebo; after 16 weeks of treatment, a significantly greater rate of patients achieved EASI-75 and IGA0/1 on 150 mg/Q2W (IGA0/1: 21.4%; EASI75: 28.6%) and 300 mg/Q2W (IGA0/1: 17.5%; EASI-75: 27.8%) with no need of rescue therapy, versus placebo (IGA0/1: 4.3%, EASI-75: 6.4%) (64). Ongoing trials are evaluating the treatment of tralokinumab for AD pediatric patients [NCT05388760] (65).

Lebrikizumab, is a fully humanized IgG4 antibody, acts by binding IL-13 in a different non–receptor-binding domain, preventing IL-4Rα/IL13Rα1 heterodimerization and downstream signaling (58). In a double-blinded, placebo-controlled, multicenter, phase 3 clinical trial, 145 subjects have been randomized to subcutaneous lebrikizumab and 66 to placebo Q2W in combination with TCS. At week 16, IGA (0.1) was achieved by 60 (41.2%) patients in the lebrikizumab plus TCS group vs. 15 (22.1%) receiving placebo plus TCS (*p* = 0.01); corresponding proportions of patients achieving EASI-75 were 69.5% (101/145) vs. 42.2% (28/66) (*p* < 0.001) (66). The most frequently reported treatment-emergent adverse events were headache (7 [4.8%]), conjunctivitis (7 [4.8%]), injection site reactions (4 [2.8%]), hypertension (4 [2.8%]), and herpes infection (5 [3.4%]) (62). Full trial results on lebrikizumab are still lacking (67, 68).

A phase 2, randomized, placebo-controlled, study evaluating the efficacy and safety of cendakimab (CC-93538) an anti- IL-13, in the treatment of moderate to severe AD, is still ongoing (69). Two hundred and fourteen patients have been enrolled in 3 different dosing regimens groups and in a placebo group (69). To date, no data are available (69).

Interestingly, it has been reported that the increased expression of IL-13 is linked with an optimal clinical response to dupilumab, while, on the other hand, non-responders patients showed to express less IL-13 and relatively greater levels of Type 1 and 3 cytokines. Hence, IL-13 levels may represent a predictor of response to IL-13 inhibitors (70).

IL-13 also showed to be a potential target even in other skin diseases, indirectly suggesting a pathogenetic role of its pathway. Indeed, dupilumab has been proposed as potentially safe and effective therapeutic option for other diseases, such as Grover disease. Evidence supporting this finding includes the fact that IL-4 infusions may induce Grover disease and that the immunophenotype of the immune cells infiltrating Grover disease demonstrate a TH2 cytokine phenotype (71).

Finally, some authors proposed the use of anti-IL-13 vaccination which may have the potential of outperforming monoclonal antibody-based approaches (72, 73).

## Conclusion

Current evidence suggest IL-13 as a crucial cytokine AD pathogenesis, supporting its significant contribution in mediating several clinical features, including skin inflammation and pruritus. Therefore, IL-13 is considered a valid target for AD. Indeed, IL-13 inhibitors such as tralokinumab and lebrikizumab seem to be a possible treatment for patients with moderate-to-severe AD, with good safety and efficacy profiles. However, further investigations will be worthy to clarify the position of these treatments in the therapeutic ladder of AD.

## Authorship

All named authors meet the International Committee of Medical Journal Editors (ICMJE) criteria for authorship for this article, take responsibility for the integrity of the work as a whole, and have given their approval for this version to be published.

## Author contributions

MN, CP, FV, AR, and GF: conceptualization. CP, MN, FV, and AR: methodology. MN, FV, and AR: software. CP, MN, FV, AR, and GF: formal analysis. MN, CP, FV, and AR: data curation and writing–original draft preparation. CP: writing–review and editing. GF: visualization. GF and CP: supervision. All authors have read and agreed to the published version of the manuscript.

## Conflict of interest

MN served as advisory board member and consultant, and has received fees and speaker’s honoraria for Sanofi, Abbvie, Leo Pharma, Lilly, Amgen. GF is a member of the journal’s Editorial Board, and has been principal investigator in clinical trials sponsored by and/or has received personal fees from AbbVie, Abiogen, Almirall, Celgene, Eli-Lilly, Leo Pharma, Novartis, Sanofi, and UCB. CP acted as investigator, speaker, consultant, and advisory board member for AbbVie, Eli Lilly, Novartis, Pfizer, and Sanofi.

The remaining authors declare that the research was conducted in the absence of any commercial or financial relationships that could be construed as a potential conflict of interest.

## Publisher’s note

All claims expressed in this article are solely those of the authors and do not necessarily represent those of their affiliated organizations, or those of the publisher, the editors and the reviewers. Any product that may be evaluated in this article, or claim that may be made by its manufacturer, is not guaranteed or endorsed by the publisher.
